# Does Technostress Increase R&D Employees' Knowledge Hiding in the Digital Era?

**DOI:** 10.3389/fpsyg.2022.873846

**Published:** 2022-06-03

**Authors:** Zhengang Zhang, Baosheng Ye, Zhijun Qiu, Huilin Zhang, Chuanpeng Yu

**Affiliations:** ^1^School of Business Administration, South China University of Technology, Guangzhou, China; ^2^Guangzhou Institute of Digital Innovation, Guangzhou, China; ^3^Economics Department, City University of New York, New York, NY, United States; ^4^Department of Tourism Management, South China University of Technology, Guangzhou, China

**Keywords:** knowledge hiding, techno-invasion, techno-insecurity, techno-complexity, techno-overload, techno-uncertainty, work exhaustion, workplace friendship

## Abstract

Technostress as an antecedent factor exploring knowledge hiding continues to be seldomly discussed in the digital era. Based on the job demand-resource theory, this article introduces work exhaustion as a mediator variable and constructs a model that the five sub-dimensions of technostress (i.e., overload, invasion, complexity, insecurity, and uncertainty) affect knowledge hiding for R&D employees. Similarly, this study analyzes the moderation of workplace friendship as the resource buffering effect. Based on data from the 254 questionnaires of the two-stage survey, empirical results show that: (1) Techno-invasion, techno-insecurity, and techno-complexity have significant positive effects on work exhaustion, and techno-invasion has the greatest effect. However, techno-overload and techno-uncertainty have no significant relationship with work exhaustion. (2) Work exhaustion plays a mediating role in the relationships between the three aspects of technostress (techno-invasion, techno-insecurity, techno-complexity) and knowledge hiding; However, its mediating effects are insignificant in the relationships between the two aspects of technostress (techno-overload and techno-uncertainty) and knowledge hiding. (3) Workplace friendship negatively moderates the relationships between the two aspects of technostress (techno-invasion and techno-insecurity) and work exhaustion, leading to less knowledge hiding. Nonetheless, its negative moderation for the relationships between the two aspects of technostress (techno-overload and techno-uncertainty) and work exhaustion are insignificant. Empirical results further show that workplace friendship positively moderates the relationship between techno-complexity and work exhaustion.

## Introduction

Knowledge management is important for R&D employees to carry out innovative activities, and enterprises continue to take measures to promote knowledge sharing among R&D employees (Serenko and Bontis, 2016; Wu and Chen, 2021). Knowledge sharing is one of the core processes of knowledge management, which is “a relational act based on a sender-receiver relationship that incorporates communicating one's knowledge to others as well as receiving others knowledge” (Foss et al., 2009, p. 873). This definition indicates that knowledge-sharing behavior consists of both donating knowledge and collecting knowledge (De Vries et al., 2006). Knowledge donating emphasizes that the knowledge sender communicates personal intellectual capital to others, while knowledge collecting emphasizes that the knowledge recipient actively consults others for their intellectual capital (De Vries et al., 2006). However, organizations do not “own” the employees' intellectual assets, and there are various counterproductive knowledge behaviors where employees fail to share their knowledge, facing the problem of knowledge-hiding behavior (Connelly et al., 2019; Bari et al., 2020). Knowledge hiding is defined as an act whereby “a person deliberately tries to conceal the knowledge required by others” (Connelly et al., 2012). That is, when employees are asked to provide knowledge by their colleagues, they deliberately conceal knowledge rather than share it. Research shows that knowledge hiding has serious negative outcomes (Connelly et al., 2019; He et al., 2021a). For example, knowledge hiding impedes the exchange and flow of knowledge within an organization (Connelly et al., 2012) and reduces employees' levels of organizational commitment and innovation (Serenko and Bontis, 2016), to name a few. Especially, knowledge hiding will have a great negative effect on the work for the group of the R&D employees in terms of the knowledge-intensive industry (Jha and Varkkey, 2018). How to reduce the knowledge hiding behavior of the R&D employees has become an important research issue. Scholars have researched to explore the antecedents of knowledge hiding behavior from various theoretical perspectives (Zhao et al., 2019; Khalid et al., 2020; Yao et al., 2020a,b; Zhao and Jiang, 2021). These studies about knowledge hiding antecedents can be classified as three aspects: individual factors, team and interpersonal factors, and organizational factors (Sofyan et al., 2021). However, prior research on R&D employees' knowledge hiding behavior from the perspective of digital technology stress remains scarce. Hence, exploring new insights into the link between technology stress and knowledge hiding is needed.

Digital technology has created a new business environment and opened up a new path for enterprise development (Yu et al., 2020b). The outbreak of COVID-19 accelerates the digital transformation process (Kudyba, 2020). For example, *WeCom*, Tencent's dedicated product for business communication and office collaboration, reached 180 million active users with more than 10 million companies and organizations in 2021. Despite enjoying the positive role of digital technology, employees also bear some negative impacts (Saleem et al., 2021). Technostress refers to a kind of psychological state caused by a person's inability to cope with new ICTs in a healthy way, which consists of five dimensions, including techno-overload, techno-invasion, techno-insecurity, techno-complexity, and techno-uncertainty (Tarafdar et al., 2007, 2015). A growing body of literature has shown that technostress can cause a host of negative outcomes (Bondanini et al., 2020), such as reduced performance (Ragu-Nathan et al., 2008; Tarafdar et al., 2015; Yener et al., 2020), decreased organizational commitment and job satisfaction (Ragu-Nathan et al., 2008), discontinued use of social networking services (Maier et al., 2015), as well as increased turnover intention, work-family conflict, and family burnout (Harris et al., 2021). Surprisingly, however, the potential effect of technostress on knowledge-related behavior has received relatively little attention. It is of great importance to fill such an unexplored gap in the literature, as knowledge has become an essential prerequisite for organizational survival and success in the knowledge-based economy (Peng, 2013). Consistent with the extensive evidence that technostress leads to a variety of dysfunctional behaviors (La Torre et al., 2019), and the emerging research calls for more attention for the triggers of technological turbulence on intra-organizational hiding (Arias-Perez and Velez-Jaramillo, 2021), we infer that technostress may also evoke knowledge hiding and propose the research question that does technostress increase R&D employees' knowledge hiding? Specifically, how do five dimensions of technostress individually influence knowledge hiding?

Based on the job demand-resource (JD-R) theoretical framework, this study explores *how* five sub-dimensions of technostress affect R&D employees' knowledge hiding and examines *when* to buffer the negative effect of technostress by introducing work exhaustion as a mediator variable and workplace friendship as a moderator. First, this paper used Vosviewer software to summarize the existing research and constructs a research framework model on the basis of JD-R theory. Second, this paper puts forward some hypotheses, including the direct and indirect effects and moderation effects between technostress and knowledge hiding. Third, this paper thoroughly introduces the research methods, including questionnaires, measurement, and statistical analysis tools. Fourth, this paper describes the analysis process and results of the study. Finally, this paper discusses the conclusions, theoretical and practical significance of the conclusions, and puts forward the future research direction. Our study results provide the reference for enterprises to reduce the knowledge hiding behaviors of the R&D employees.

## Literature and Theoretical Foundation

### Literature Review

Knowledge hiding can be understood as consisting of three different facets, including evasive hiding, playing dumb, and reasonable hiding (Connelly et al., 2012). Evasive hiding means that the knowledge hider provides incorrect knowledge to the requester or pretends to agree to help but actually attempts to delay; playing dumb means that the knowledge hider pretends not to know or understand the problem of the knowledge requestor; reasonable hiding means that the knowledge hider explains to the requester why the required knowledge is not provided, such as being required to keep the required knowledge confidential (Connelly et al., 2012). According to the above statements, evasive hiding and playing dumb often involve high deception (Connelly and Zweig, 2015; Zhao et al., 2019; Peng et al., 2021), and this study emphasizes the negative effects of knowledge hiding on individuals and organizations. Although people may pretend to hide their knowledge through reasonable hiding, it also has many benefits for an enterprise in terms of carrying out the enterprise's secret system, obeying the superior's order or the department rule, and its deception is low (Connelly et al., 2012). In addition, a large number of scholars believe that reasonable hiding is different in nature from evasive hiding and playing dumb (Zhao et al., 2019; Peng et al., 2021). Hence, this study utilized only two dimensions of knowledge hiding, namely, evasive hiding and playing dumb.

**Figure 1:**
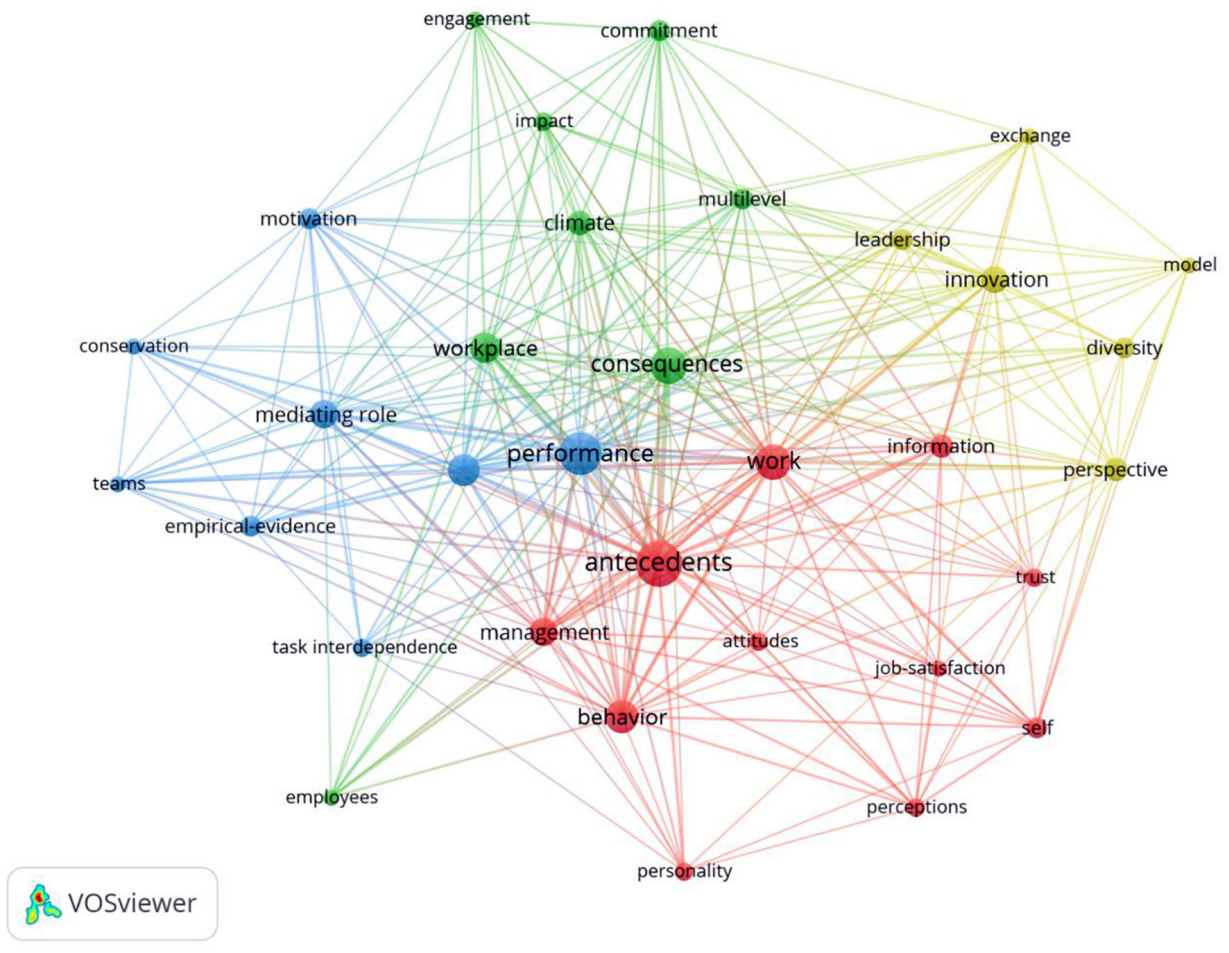
An overview of the knowledge hiding literature. The database is “web of science core collection,” and the retrieval condition is “topic = knowledge hiding.” To ensure data accuracy, we carefully selected studies that fit the definition given by Connelly et al. (2012) and retained research articles, review articles, and online publications. This process yielded 182 articles (Retrieved on December 1, 2021).

Since the concept of knowledge hiding was proposed, many studies have explored the antecedents and consequences of this behavior, and the consequences of knowledge hiding have focused on the impact on performance and innovation (see Figure 1, each color represents a cluster).

First, literature cluster 1 focuses on the antecedent variables for knowledge hiding. Specifically, the frequencies of antecedents (frequency = 24), work (frequency = 14), and behavior (frequency = 12) in cluster 1 are higher (see Table 1), which show that the cluster focuses on the antecedent variables of knowledge hiding. These antecedents for knowledge hiding can arise from the perspective of individual, interpersonal/team, and organizational levels (Sofyan et al., 2021). The individual factors can be seen in aspects such as role stress (Zhao and Jiang, 2021), compulsory citizenship behavior (He et al., 2019), and feelings of psychological ownership (Peng, 2013). The interpersonal factors include aspects such as workplace bullying (Yao et al., 2020b) and ostracism (Serenko and Bontis, 2016), and the team level factors involve aspects such as team motivational climate (Černe et al., 2014). In addition, the organizational-level determinants include aspects such as leader-member exchange (Zhao et al., 2019) and organizational knowledge culture (Serenko and Bontis, 2016). For example, Zhao and Jiang (2021) analyzed the impact of role stress on knowledge hiding from the perspective of social network theory and role theory. Zhao and Liu (2021) explored the perceptions of corporate hypocrisy on knowledge hiding on the basis of social cognitive theory.

**Table 1:** Clustering analysis of knowledge hiding literature (Clusters 1–4).

**Label**	**Cluster**	**Occurrences**	**Label**	**Cluster**	**Occurrences**
Antecedents	1	24	Consequences	2	15
Work	1	14	Workplace	2	10
Behavior	1	12	Climate	2	7
Management	1	9	Commitment	2	5
Information	1	6	Multilevel	2	5
Self	1	5	Impact	2	4
Attitudes	1	4	Employees	2	3
Perceptions	1	4	Engagement	2	3
Personality	1	4			
Trust	1	4			
Job satisfaction	1	3			
Performance	3	20	Innovation	4	8
Moderating role	3	11	Perspective	4	6
Mediating role	3	9	Diversity	4	5
Empirical evidence	3	5	Leadership	4	5
Motivation	3	5	Exchange	4	3
Task	3	4	Model	4	3
interdependence					
Conservation	3	3			
Teams	3	3			

Second, literature cluster 2 focuses on the consequences of knowledge hiding behavior, which can be seen from the high frequency of keywords occurrence, such as consequences (frequency = 15), workplace (frequency = 10), and climate (frequency = 7; see Table 1). Current research explores the consequences of knowledge hiding mainly from the individual and team aspects (He et al., 2021a). In terms of individual aspects, the existing research has examined the effects of knowledge hiding on individual job performance, psychological status and attitude, workplace behavior, and supervisor-subordinate/coworker relationships (He et al., 2021b). For example, Bari et al. (2020) analyzed the influence of different dimensions of knowledge hiding on employee silence. In terms of team aspects, prior studies have found that knowledge hiding has significant negative effects on team performance (Zhang and Min, 2019), team creativity (Fong et al., 2018), team viability (Wang et al., 2018), team learning, and absorptive capability (Fong et al., 2018; Zhang and Min, 2019). For example, Zhang and Min (2019) investigated the effect that knowledge hiding influences project team performance through team learning.

Third, literature cluster 3 focused on the mechanism and moderation that knowledge hiding affects performance, which is shown in the higher frequency of performance (frequency = 20), moderating role (frequency = 11), and mediating role (frequency = 9; see Table 1). For example, Ain et al. (2021) examined the relationship between knowledge hiding and extra-role performance, considering the mediating role of emotional exhaustion and the moderating role of political skills. Khoreva and Wechtler (2020) explored the different facets of knowledge hiding on individual-level job performance as well as the mediating role of employee wellbeing.

Finally, literature cluster 4 focused on the impact of knowledge hiding on innovation, which is displayed by the higher frequency of innovation (frequency = 8), perspective (frequency = 6), and diversity (frequency = 5; see Table 1). The main logic is that knowledge hiding reduces the positive effect of knowledge diversification for innovation. For example, Zhang and Min (2019) explored the impact of knowledge hiding on team product R&D innovation performance. Bogilovic et al. (2017) used social exchange theory and social classification theory to put forward that individual knowledge hiding is negatively related to individual creativity.

Unfortunately, following the above analysis, studies rarely deal with the analysis of factors concerning the technology stress that we believe can also become triggers for knowledge hiding. With the acceleration of digital transformation, more and more enterprises use digital technology (Arias-Perez and Velez-Jaramillo, 2021), and it also promotes employee technostress because of the increased work overload, excessive technology dependence, demands for enhanced productivity, and a constant need to adapt to emerging ICT (information and communications technologies) applications, functionalities, and workflows (Srivastava et al., 2015). Existing literature does not explain the impact of technostress on R&D employee knowledge hiding, therefore further studies are required.

### Research Model

**Figure 2:**
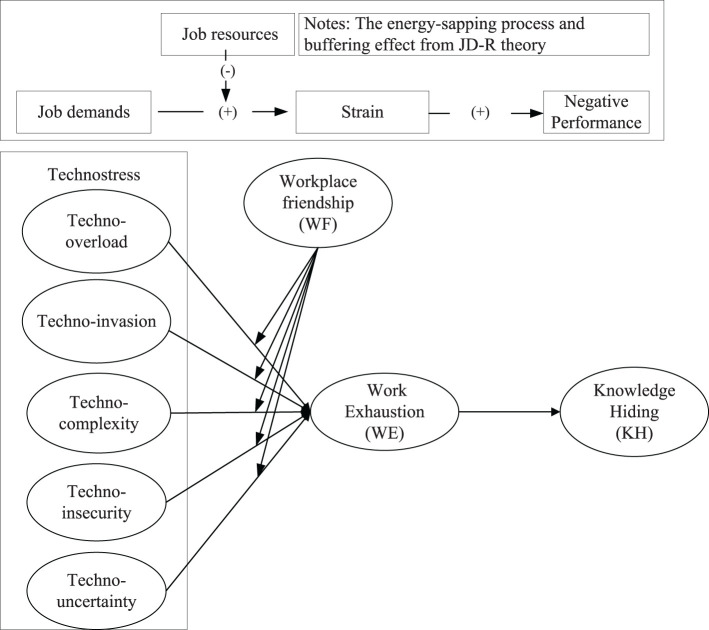
Conceptual framework.

We construct a model that explains *why* and *when* technostress increases R&D employee knowledge hiding following the JD-R theory. As for the same research question, we may obtain different conceptual models from different theoretical perspectives. In this study, we derived a conceptual model on the basis of the JD-R theory (see Figure 2) to answer the research question of “Does technostress increase R&D employee knowledge hiding in the digital era?” The entire logic is as follows.

First, the premise of applying the JD-R theory is to identify clearly two broad categories of work conditions, namely, job demands and job resources. In the JD-R theory, job demands are defined as “those aspects of the job that require individual physical and/or psychological efforts or costs” (Demerouti et al., 2001; Bakker and Demerouti, 2017). Simply, job demands are the “negative aspects of the job” that consume individual energy at work, such as work overload, time pressure, and challenging demands, among others (Ingusci et al., 2021). Considering that technostress requires physical and psychological efforts from the R&D employee, we consider technostress as a job demand. Job resources are “those aspects of the job that are functional in achieving work goals, reduce job demand, or stimulate personal growth, learning, and development” (Demerouti et al., 2001; Bakker and Demerouti, 2017). They are the “positive aspects” in the work, and the examples of job resources include work autonomy, feedback relating to performance, and social support (Ingusci et al., 2021). The R&D employees with a high level of workplace friendship can obtain support and help from their colleagues to promote the achievement of work goals and reduce the psychological and physiological costs associated with job demands (Chang et al., 2016). Therefore, we regard workplace friendship as a kind of job resource.

Second, the JD-R theory provides an explanation logic for the research question of “does technostress increase R&D employee knowledge hiding in the digital era?” after identifying the theoretical premise. Following the energy-sapping process of the JD-R theory, individuals are prone to consume resources due to high job demands (Bakker and Demerouti, 2007). The insufficient resources (or exhaustion) will easily increase the negative performance (Bakker and Demerouti, 2007). The strong technostress represents the high job demands to respond to digital technology. Under high job demands, the R&D employees will exhaust resources to cope with the higher technostress (Santuzzi and Barber, 2018), and the insufficient resources will increase knowledge hiding (Montani et al., 2020), which is a kind of a negative outcome.

Moreover, the JD-R theory provides a “resource buffering” hypothesis (Bakker and Demerouti, 2017), which can be used to explain the moderation of workplace friendship. The job resources can buffer the high job demands on the loss of psychological resources. That is, job resources can mitigate the negative impact of job demands on employees (Bakker et al., 2005). According to this theoretical logic, workplace friendship, as a working resource, may reduce the effect of technostress on work exhaustion and further reduce the mediation relationships of “technostress (including 5 dimensions)-work exhausting-knowledge hiding.” The introduction of workplace friendship can be understood primarily through the following reasons. The moderating effect is considered to indicate that, under certain conditions, the main effect relationship may be insignificant or low. Hence, if workplace friendship has a buffering effect, it can interfere with the relationship between technostress and work exhaustion, helping the enterprise better reduce the effects of technostress on knowledge hiding.

Based on the JD-R theory, we explore the mechanism of technostress, influencing knowledge hiding through work exhaustion and analyze the buffering effect of workplace friendship. Under the background of the digital age, this research contributes new findings on the effects of technology stress and connects the seams with knowledge hiding.

## Hypothesis Development

### Technostress and R&D Employee Work Exhaustion

Work exhaustion is defined as “long-term, intense physical, emotional, and cognitive stress resulting from prolonged exposure to specific working conditions or stressors” (De Merouti et al., 2003). According to the JD-R theory, when faced with higher job demands, employees tend to consume resources to respond (Bakker and Demerouti, 2017). As resources are depleted, employees are likely to fall into a state of insufficient resources. When R&D employees cannot obtain sufficient resources to meet their job demands, they will fall into a state of work exhaustion. The relationships between the five dimensions of technostress and work exhaustion are as follows.

Techno-overload means that employees work more, longer, and faster due to digital technology use (Tarafdar et al., 2007; Shadbad and Biros, 2020). That is, employees work quickly on a tight schedule and handle huge loads (Ragu-Nathan et al., 2008). First, digital technology allows the R&D employees to accomplish work faster and more efficiently, and these processes require them to complete additional work in the same amount of time (Ingusci et al., 2021). An increase in work intensity leads to an increase in resource consumption for R&D employees, and as a result, the lack of resources will lead to work exhaustion. Second, digital technology has brought more electronic information through the email, Internet or phone, among others. The increasing electronic information compels employees to feel a sense of “information fatigue” (Barley et al., 2011). The long hours and frequent responses to electronic information consume the personal resources of the R&D employees, ultimately leading to work exhaustion.

Techno-invasion is when digital technology intrudes the employee's personal life and interferes with work-family balance as employees can be contacted anytime and anywhere (Ragu-Nathan et al., 2008). First, the use of digital technology has led to nearly constant contact, leading the R&D employees to feel that they are never free of digital technology, and their time and personal spaces have been invaded (Ragu-Nathan et al., 2008). The evaporation of the balance between individuals' private lives and work might also lead to a feeling of work exhaustion (Yener et al., 2020). Second, the large amounts of work-related emails or information force employees' life time and space to be allocated to work (Sofyan et al., 2021). Under this condition, the R&D employees lose their passion for work, which eventually lead to work exhaustion.

Techno-complexity refers to the circumstances in which an employee is inexpert in using digital technology and needs to spend time and energy to gain knowledge (Tarafdar et al., 2007; Shadbad and Biros, 2020). First, the development of digital technology has brought additional complex knowledge and skills, and learning the complexity of concepts and terminology makes the R&D employees feel overwhelmed and frustrated (Tarafdar et al., 2015). Then, the R&D employees easily feel work exhaustion. Second, understanding new concepts and terms requires an enormous amount of time and effort for the R&D employees, which allows for less rest time outside work. The lack of rest exhausts the resources of R&D employees, which ultimately affects their working state and brings them into work exhaustion.

Techno-insecurity is when the employees feel they may lose their jobs either by computerization of job tasks or having less knowledge about digital technology than others (Tarafdar et al., 2007). First, digital technology has made the work of the R&D employees fungible, and the use of artificial intelligence and robots can carry out to work, which substitutes for employees (Arias-Perez and Velez-Jaramillo, 2021). The unsafe feeling of job substitution will lead the R&D employees to work exhaustion. Second, if the employees have no solid knowledge and skills in digital technology, then they will face the threat of unemployment. The fear of unemployment will compel employees to be in a long-term negative mood and eventually lead to work exhaustion.

Techno-uncertainty refers to the constant changes and upgrades in digital technology that puts new pressure on employees (Tarafdar et al., 2007). Under this situation, the rapid transformation of digital technology has reduced the morale of the R&D employees (Tarafdar et al., 2015), and it puts forward new requirements for employees' work content and knowledge structure (Shadbad and Biros, 2020). These uncertainties threaten employees' diminished control over their jobs, selves, and technology, consequently increasing work exhaustion.

To sum up, the R&D employees experience five dimensions of technology stressors, which consume their personal resources and trap them in work exhaustion. Hence, hypotheses 1a−1e are proposed:

Hypothesis 1: Techno-overload (1a), techno-invasion (1b), techno-complexity (1c), techno-insecurity (1d), or techno-uncertainty (1e) are positively correlated with R&D employee work exhaustion.

### Work Exhaustion and R&D Employees' Knowledge Hiding

According to the JD-R theory, when an individual's psychological resources are insufficient, inducing negative work results is easy. Work exhaustion, which is considered a low psychological resource state, can reduce the employee's enthusiasm for work and convey unsafe signals (Zhao and Jiang, 2021). In addition, work exhaustion may cause employees to feel physically and mentally exhausted, lack of purpose and morale, and indifferent to organizational affairs (Zhao and Jiang, 2021). Then, the R&D employees would be less likely to have the will, time, and energy to deal with knowledge requests (Guo et al., 2021). In this case, employees may choose to hide knowledge when facing knowledge requests from other people to avoid further consumption of personal resources. In other words, work exhaustion increases the likelihood of R&D employees' knowledge hiding behavior. Based on this assumption, hypothesis 2 is proposed.

Hypothesis 2: Work exhaustion is positively correlated with R&D employees' knowledge hiding.

### Mediating Effect of Work Exhaustion

The JD-R theory indicates when employees are faced with high job demands, these demands will lead to work exhaustion and, in turn, result in negative work outcomes (Bakker and Demerouti, 2017). Following this logic, if the R&D employees perceive high technostress, it will lead to a state of work exhaustion and then increase knowledge-hiding behavior. In the case of higher technostress, the R&D employees tend to expend their personal resources to relieve stress. The more personal resource consumption increases the likelihood of work exhaustion, leading to knowledge hiding behavior to avoid further consumption of personal resources for sharing knowledge. Hence, work exhaustion acts as a mediator between technostress and knowledge hiding. Considering hypotheses 1a−1e and hypothesis 2, the logic of work exhaustion playing a mediator between the five dimensions of technostress and knowledge hiding is as follows.

If the R&D employees experience techno-overload, they have to deal with heavy work in a short time and frequently respond to electronic information (Tarafdar et al., 2007; Ragu-Nathan et al., 2008). On the one hand, the R&D employees need to deal with multiple tasks simultaneously *via* digital technology. The processing of multiple tasks require the R&D employees to invest additional resources, and insufficient resources can easily lead to work exhaustion. The R&D employees surrounded in work exhaustion are more inclined to hide knowledge to prevent further depletion of resources. On the other hand, the techno-overload also results in “information fatigue” for the R&D employees (Barley et al., 2011). That is, the R&D employees have received a large amount of information by using digital technology. Additionally, identifying relevant information and setting practical cut-offs and priorities regarding new information become difficult. In the process, the R&D employees consume a substantial amount of psychological resources and easily fall into work exhaustion. Then, they can easily choose to hide their knowledge to prevent further depletion of the resources.

When the R&D employees feel the stress of techno-invasion, they have to work anytime and anywhere due to digital technology (Ragu-Nathan et al., 2008; Tarafdar et al., 2015). They do not get adequate rest and easily fall into work exhaustion. The R&D employees who are caught in exhaustion can easily choose to hide knowledge to reduce further consumption of resources. Furthermore, the R&D employees perceive that they are working all day and have no private space (Srivastava et al., 2015), which easily blurs the boundary between work and home and makes the employees feel disgusted with their work in terms of work-family conflict (Gaudioso et al., 2017; Sofyan et al., 2021), leading them to choose to hide their knowledge.

The complexity of digital technology induces the R&D employees' need to invest time and energy to seek further knowledge (Shadbad and Biros, 2020). The more resource consumption to learn can easily lead to the R&D employees falling into work exhaustion. The R&D employees feel very tired of their work and are reluctant to share knowledge with other employees. Furthermore, owing to the techno-complexity, the R&D employees are unlikely to use technology to work and thus fall into work exhaustion. In these circumstances, the R&D employees tend to hide their knowledge.

Under the insecurity of digital technology, the R&D employees are worried that digital technology will replace their jobs. This kind of worry can easily lead to a sense of tension and anxiety, which, in turn, leads to work exhaustion. The R&D employees with work exhaustion are likely to hide their knowledge. In addition, if the R&D employees lack continuous learning ability, they are worried that employees with higher digital skills will replace their jobs (Shadbad and Biros, 2020). This peer pressure among colleagues increases the psychological burden, resulting in work exhaustion. Employees are reluctant to share their knowledge and choose to hide it.

When the R&D employees perceive high technological uncertainty, it puts forward new requirements for the employees' work content and knowledge structure because the pace of digital transformation is extremely fast (Shadbad and Biros, 2020). These uncertainties threaten employees' diminished control over their jobs, selves, and technology, increasing work exhaustion. Under the situation of work exhaustion, the R&D employees would choose to hide their knowledge to express their dissatisfaction. Based on the above propositions, hypotheses 3a−3e are proposed.

Hypothesis 3: Work exhaustion acts as a mediator between the five aspects of technostress [techno-overload (3a), techno-invasion (3b), techno-complexity (3c), techno-insecurity (3d), techno-uncertainty (3e)] and knowledge hiding.

### Moderating Effect of Workplace Friendship

The JD-R theory holds a hypothesis of “buffering effect,” which indicates that job resources buffer the relationships between job demands and work exhaustion. For employees with several job resources, the relationships between job demands and work exhaustion will be weak (Bakker and Demerouti, 2017). In this study, we regard workplace friendship as a job resource and technostress as a job demand, assuming that workplace friendship plays a moderating role between technostress and work exhaustion.

Workplace friendship is “a kind of non-coercive interpersonal relationship formed on the basis of voluntary principle” (Wright, 1984). It consists of two dimensions, including workplace friendship opportunity and workplace friendship prevalence (Nielsen et al., 2000), which can be understood that mutual trust, commitment, and shared interests or values exist (Berman et al., 2002). Workplace friendship is considered in the study as a job resource that buffers the depletion of staff resources due to high job demands. For employees with a high level of workplace friendship, maintaining friendly interpersonal relationships with colleagues is possible in the workplace, which can meet the emotional needs of individuals in the workplace. At the same time, a high level of workplace friendship can increase their enthusiasm and confidence in coping with work, which can help them continue working or reduce stress more quickly in the face of technostress. A high level of workplace friendship means positive relationships, which creates a pleasant and relaxing work environment and helps mitigate the effects of technostress on work exhaustion.

Under higher workplace friendship, the R&D employees obtain other emotional resources provided by the enterprise and they are willing to invest more energy for the enterprise. Then the techno-overload is less likely to cause work exhaustion. Likewise, workplace friendship satisfies the emotional, belonging needs of the R&D employees (Yu et al., 2021) and can compensate for the lack of time with family members caused by techno-invasion. Furthermore, high workplace friendship can encourage the R&D employees to learn new knowledge and skills from one another, reducing the relationship between techno-complexity and work exhaustion. In addition, workplace friendship creates a relaxed, enjoyable, and harmonious work environment (Yu et al., 2021), which creates a feeling of safety and optimism among the R&D employees about their tasks, reducing the relationship between techno-insecurity and work exhaustion. Finally, workplace friendship allows the R&D employees to gain additional support from their colleagues (Yu et al., 2021). They can acquire further technical information and update their knowledge structure in time, better weakening the relationship between techno-uncertainty and work exhaustion. Based on the above analysis, we propose hypotheses 4a–4e.

Hypothesis 4: Workplace friendship will negatively moderate the relationships between the five dimensions of technostress [techno-overload (4a), techno-invasion (4b), techno-complexity (4c), techno-insecurity (4d), techno-uncertainty (4e)] and work exhaustion. When workplace friendship is high, the relationships between the five dimensions of technostress [techno-overload (4a), techno-invasion (4b), techno-complexity (4c), techno-insecurity (4d), techno-uncertainty (4e)] and work exhaustion will be weaker.

## Methods

### Questionnaire Design, Sample, and Data Collection

Since technostress, work exhaustion, and workplace friendship are the psychological perception variables that are difficult with second-hand data to measure, this article uses self-reported scales to measure them following the guidance of Zhao and Jiang (2021). In addition, a prior study indicated that this method is reasonable (Huo et al., 2016). The data collected at the same time period may lead to common method biases (Podsakoff et al., 2003), causing the illusory correlations in the estimation results. Therefore, the data collections were conducted at two time periods to reduce the potential impact of common method biases. Given the difficulty of two-time data collection, we chose a professional research firm to assist us for two reasons: First, choosing a professional research firm is preferable because the scattered R&D employees could be accessed in a short time period and at a low cost (Khan et al., 2020). Second, the firm has an extensive list of samples and can minimize missing values by using some techniques, because the respondents were not allowed to proceed to the next question if they did not answer the current one and thus ensured high survey quality.

The respondents were the R&D employees, and the survey period was from November to December 2021. In Time 1, we collected the variables including techno-overload, techno-invasion, techno-complexity, techno-insecurity, techno-uncertainty, friendship opportunity, friendship prevalence, and related control variables. A total of 455 questionnaires were distributed to the R&D employees, and 355 valid questionnaires were returned (78.0%). In Time 2, the respondents who had completed the valid questionnaire in Time 1 were required to assess the level of work exhaustion and knowledge hiding (including evasive hiding and playing dumb). A total of 254 respondents returned their valid questionnaires (71.5%). Table 2 presents the details of the sample.

**Table 2:** Description of the sample (*N* = 254).

**Profile of respondents**	**Class**	**Frequency**	**%**
Gender	Female	85	33.5
	Male	169	66.5
Age (in years)	<25	41	16.1
	25–35	196	77.2
	>35	17	6.7
Education	Bachelor degree	199	78.3
	Master and Doctor degree	55	21.7
Rank	Manager	48	18.9
	Supervisor	93	36.6
	Employee	113	44.5
Working tenure (in years)	<3	53	20.9
	3–5	95	37.4
	>5	108	41.7

### Measurement

The measurement scales were derived from existing literature, and we made certain modifications according to our study context. Guided by the practice of relevant literature (Yu et al., 2020a), we designed the scales following three steps. Step one was to collect relevant scales, considering some criteria including the degree of match with the research object, the reliability and validity, and the cited frequency of the scale. Step two was to use the “back translation” method to provide the respondents with scales in Mandarin Chinese. That is, for the English scales, we first invited two researchers with good English to translate the items into Mandarin Chinese, and then two other researchers translated it from Mandarin Chinese to English to check the accuracy of translation. Step three was to consult two scholars who specialized in organizational behavior research and two managers from the human resources management department to listen to their opinions for the scale items. While not changing the basic structure of the translated scale, we revised and improved the item expressions following the opinions of the scholars and the managers and finally formed the measurement scale. All the measures were rated using a seven-point Likert-type scale from “1 = totally disagree” to “7 = totally agree.” The specific sources of the scales are as follows:

Knowledge hiding, which includes two dimensions of “evasive hiding” and “playing dumb,” is derived from Connelly et al. (2012). In our study, we measured the influence of the R&D employees who deliberately pretend to hide knowledge requested by others, emphasizing the negative impact of knowledge hiding behavior on the enterprise. Hence, we excluded the dimension of reasonable hiding that may somewhat have a positive effect on the enterprise to measure knowledge hiding in our analysis. The scales of evasive hiding (four items) and playing dumb (four items) are also used by Zhao et al. (2019) and Arias-Perez and Velez-Jaramillo (2021).

The technostress scale is adapted from Tarafdar et al. (2015), including five dimensions. Techno-overload measures the use of digital technology to have increased work requirements and workload of R&D employees. Techno-invasion measures the use of digital technology to have invaded private life, blurred the work-family boundary of R&D employees, and aggravated work-family conflict. Techno-complexity measures the need for R&D employees to invest extra time and energy in learning and mastering digital technology or encounters the difficulties or frustrations in learning new digital technologies. Techno-insecurity measures R&D employees' perception of their work possibilities which is replaced by technology or replaced by employees with higher digital technology literacy. Techno-uncertainty measures the continuous acceleration of digital technology updates, which makes it difficult for R&D employees to respond.

A five-item scale derived from Moore (2000) was used to access the work exhaustion construct. It measures the negative psychological feelings of the R&D employees who feel that their psychological resources are insufficient to cope with work needs.

Workplace friendship is revised with reference to the scale of Nielsen et al. (2000) in the Chinese context. Chinese scholars Sun and Jiao (2012) have verified the revised scale, which showed good reliability and validity. The scale included two dimensions: friendship opportunity (five items) and friendship prevalence (four items). Friendship opportunities measure the atmosphere and environment that the R&D employees perceive that the organization creates for them to build friendships at work. Friendship prevalence measures the quality and the degree of interdependence of the relationship among the R&D employees.

This study controlled the influence of relevant variables for reducing the possible alternative interpretation of the model relationship. Prior studies have suggested that gender, age, education, tenure, and position grade may affect employees' knowledge hiding behavior (Zhao and Jiang, 2021) and interfere with the explanatory power of the research model, which are controlled in this study. Gender was coded with a binary variable, 1 for male and 0 for female. Two dummy variables were set for age, and 0 represented over 35 years old, which was used as the reference group. The first dummy variable (coded as 1) represented 25–35 years old, and another dummy variable (coded as 1) represented 25 years old and under. Education was coded with a binary variable: 0 represented master and doctor's degree, and 1 represented bachelor's degree. Position grade was also set with two dummy variables: 0 represented the manager position, which was the reference group. In the first dummy variable, 1 represented the supervisor position, while 1 in another dummy variable represented the employee position.

### Statistical Analysis Tools

This article used Partial Least Squares-Structural Equation Modeling (PLS-SEM) for estimation, and the analysis tool was Smart PLS 3.0 software. Compared with the covariance-based structural equation modeling (CB-SEM), which is based on the covariance matrix, PLS-SEM can be a more suitable approach for our research model. Likewise, this method is also widely used in management research, especially some studies on knowledge hiding (Bari et al., 2020; Koay et al., 2020; Arias-Perez and Velez-Jaramillo, 2021). PLS-SEM has gradually become the mainstream questionnaire analysis tool in recent years. Specifically, the reasons for using the PLS-SEM tools in this study are as follows.

First, this article adopts the second-order construct for measuring knowledge hiding (including evasive hiding, playing dumb) and workplace friendship (including friendship opportunity and friendship prevalence). The PLS-SEM method can handle the second-order model more conveniently (Hair et al., 2017). Second, this article evaluates the moderation of latent variables for workplace friendship and analyzes the effect size of the moderation effects. Regarding the factor indeterminacy that limits CB-SEM's usefulness for moderation analyses, PLS-SEM is particularly suitable for integrating the interaction term(s) into the path mode as the method has practically no limitations (Ringle et al., 2020), which fits our study. Likewise, the f square index of the PLS-SEM software can evaluate the effect size of moderation effect. Third, based on the collected literature, we found that exploring the influence mechanism and moderating effect on knowledge hiding from the perspective of technical stress is in its infancy. Therefore, this article is an exploratory research, and we focused on the explained variance for knowledge hiding and workplace exhaustion. The PLS-SEM is based on maximizing explained variance, and the *R*^2^ is the proportion of total variance explained rather than that which is the proportion of common variance explained for CB-SEM (Hair et al., 2017), making it suitable for this study. Furthermore, PLS-SEM has lower requirements for basic statistical assumptions. Especially when the sample size is not particularly large, the estimated results are robust, which is more in line with the actual sample size of this study (the valid sample is 254).

According to the recommendations of Anderson and Gerbing (1988), we first report the results of the measurement model (the outer model), and then report the results of the structural model (the inner model).

## Analysis and Results

### Measurement Model

#### Reliability and Validity Analysis

Firstly, this paper evaluates the factor loading value of the measurement item. According to the suggestions of Hair et al. (2017), the factor loading value of the item should be >0.7. If the item is lower than 0.7 but >0.4, the composite reliability (CR) of the construct is not improved after deleting the item, and then the item can be retained. The item with very low loading (below 0.4) should always be eliminated from the construct. As shown in Table 3, the factor loading values of evasive hiding (0.792–0.876), playing dumb (0.834–0.873), techno-invasion (0.786–0.849), friendship prevalence (0.823–0.879) all satisfy the suggested criterion. For the variables of techno-complexity (Item 4), techno-insecurity (Item 2), work exhaustion (Items 4 and 5) and friendship opportunity (Items 1 and 2), their factor loading values are lower than 0.7 but >0.5. We attempted to delete the items and found that the composite reliability of variables had not been improved. Meanwhile, considering that the factor loading values are >0.5 (still in the acceptable range), they are retained in this paper. However, as for the items of techno-overload (Items 1 and 5), techno-complexity (Item 1) and techno-uncertainty (Item 3), which are lower than 0.4, four items were deleted. And these three constructs, including techno-overload, techno-complexity, techno-uncertainty, still have good composite reliability and validity (see Tables 4, 5 below), indicating deleting the items is acceptable.

**Table 3:** The constructs, items, and measurement model (*N* = 254).

**Construct and items**	**F. L**
**Evasive hiding (EH)**	
When my colleagues asked me the required knowledge, I	—
1. Agreed to help him/her but never really intended to	0.870
2. Agreed to help him/her but instead gave him/her information different from what he/she wanted	0.792
3. Told him/her that I would help him/her out later but stalled as much as possible	0.876
4. Offered him/her some other information instead of what he/she really wanted	0.796
**Playing dumb (PD)**
When my colleagues asked me the required knowledge, I	—
1. Pretended that I did not know the information	0.871
2. Said that I did not know, even though I did	0.873
3. Pretended I did not know what he/she was talking about	0.834
4. Said that I was not very knowledgeable about the topic	0.872
**Techno-overload**	
1. I am forced by this digital technology to work much faster (deleted).	0.290
2. I am forced by this digital technology to do more work than I can handle.	0.714
3. I am forced by this digital technology to work with very tight time schedules.	0.768
4. I am forced to change my work habits to adapt to new digital technologies.	0.816
5. I have a higher workload because of increased digital technology complexity (deleted).	0.141
**Techno-invasion**	
1. I spend less time with my family due to this digital technology	0.845
2. I have to be in touch with my work even during my vacation due to this digital technology.	0.786
3. I have to sacrifice my vacation and weekend time to keep current on new digital technologies.	0.795
4. I feel my personal life is being invaded by this digital technology.	0.849
**Techno-complexity**	
1. I do not know enough about this digital technology to handle my job satisfactorily (deleted).	0.355
2. I need a long time to understand and use new digital technologies.	0.869
3. I do not find enough time to study and upgrade my digital technology skills	0.822
4. I find new recruits to this organization know more about computer technology than I do.	0.691
5. I often find it too complex for me to understand and use new digital technologies.	0.840
**Techno-insecurity**	
1. I feel constant threat to my job security due to new digital technologies.	0.777
2. I have to constantly update my skills to avoid being replaced.	0.638
3. I am threatened by coworkers with newer digital technology skills.	0.788
4. I do not share my knowledge with my coworkers for fear of being replaced.	0.838
5. I feel there is less sharing of knowledge among coworkers for fear of being replaced.	0.842
**Techno-uncertainty**	
1. There are always new developments in the digital technologies we use in our organization	0.969
2. There are constant changes in computer software in our organization.	0.534
3. There are constant changes in computer hardware in our organization (deleted).	0.160
4. There are frequent upgrades in computer networks in our organization.	0.717
**Work exhaustion**	
1. I feel emotionally drained from my work.	0.807
2. I feel used up at the end of the work day.	0.820
3. I feel fatigued when I get up in the morning and have to face another day on the job.	0.811
4. I feel burned out from my work.	0.653
5. Working all day is really a strain for me.	0.633
**Friendship opportunity**	
1. I have the opportunity to get to know my coworkers.	0.631
2. I am able to work with my coworkers to collectively solve problems.	0.596
3. In my organization, I have the chance to talk informally and visit with others.	0.732
4. Communication among employees is encouraged by my organization.	0.814
5. Informal talk is tolerated by my organization as long as the work is completed.	0.725
**Friendship prevalence**	
1. I have formed strong friendships at work.	0.823
2. I socialize with coworkers outside of the workplace	0.863
3. I can confide in people at work.	0.879
4. Being able to see my coworkers is one reason why I look forward to my job.	0.796

*F.L., factor loading*.

**Table 4:** Reliability and convergent discriminant validity analysis (*N* = 254).

	**1**	**2**	**3**	**4**	**5**	**6**	**7**	**8**	**Cronbach's α**	**CR**	**AVE**
1. Knowledge hiding	**0.849**								0.907	0.954	0.720
2. Techno-overload	0.098	**0.767**							0.654	0.810	0.588
3. Techno-invasion	0.239	0.440	**0.820**						0.838	0.891	0.672
4. Techno-complexity	0.189	0.286	0.358	**0.808**					0.824	0.882	0.653
5. Techno-insecurity	0.308	0.331	0.422	0.575	**0.780**				0.839	0.885	0.609
6. Techno-uncertainty	−0.026	0.333	0.104	0.273	0.168	**0.761**			0.815	0.796	0.579
7. Work exhaustion	0.486	0.164	0.479	0.285	0.362	−0.043	**0.749**		0.801	0.864	0.562
8. Workplace friendship	−0.275	0.105	−0.097	0.074	0.000	0.398	−0.284	**0.768**	0.858	0.927	0.590

*Squared root of the average variance extracted values in bold are on the diagonal. Pearson's correlations are below the diagonal*.

**Table 5:** Heterotrait-monotrait ratio test (*N* = 254).

	**1**	**2**	**3**	**4**	**5**	**6**	**7**	**8**
1. Knowledge hiding								
2. Techno-overload	0.133							
3. Techno-invasion	0.264	0.592						
4. Techno-complexity	0.216	0.399	0.419					
5. Techno-insecurity	0.339	0.463	0.508	0.679				
6. Techno-uncertainty	0.052	0.514	0.142	0.427	0.313			
7. Work exhaustion	0.559	0.227	0.587	0.351	0.451	0.085		
8. Workplace friendship	0.330	0.184	0.151	0.143	0.139	0.468	0.354	

Secondly, the reliability, convergence, and discriminant validity were evaluated. Table 4 shows all variables of the Cronbach's α, except for techno-overload, are >0.7, and the composite reliability (CR) values are >0.819, meeting the recommended criteria value above 0.7 (Hair et al., 2017). As for the techno-overload, although its Cronbach's α is 0.654, the composite reliability (CR) value is 0.803, which is still in the acceptable range. The average variance-extracted (AVE) values of all variables are >0.562, meeting the recommended standard of >0.5.

Furthermore, the discriminant validity is measured by two approaches, including the Fornell–Larcker criterion and the heterotrait–monotrait ratio (HTMT). For the Fornell–Larcker criterion, the square root of the AVE values on the diagonal in bold characters is greater than its highest Pearson correlation with any other variables in Table 4, showing that the discriminant validity has been established (Fornell and Larcker, 1981). Considering when the factor loading values are high and their differences are slight (e.g., all indicator loadings vary between 0.6 and 0.8), the detection power of the Fornell-Larcker criterion performs very poorly (Hair et al., 2017). As a remedy, the latest HTMT is adopted to further analyze. This test method is mainly based on the logic that “the average of the monotrait-heteromethod correlations (i.e., the correlations of indicators within the same construct) should be greater than the average of the heterotrait-heteromethod correlations (i.e., the correlations of indicators across constructs measuring different phenomena)” (Henseler et al., 2015). If the ratio value between the average of the heterotrait-heteromethod correlations and the average of the monotrait-heteromethod correlations is <0.9, it indicates that there is discriminant validity between variables. Table 5 shows that the values of HTMT ratio are <0.9 (Henseler et al., 2015), which, once again, supports that the variables have good discriminant validity.

#### Common Method Variance

Since self-reported questionnaires may lead to the problem of common method variance (CMV), this paper adopted two methods, including the design of study's procedures and post-statistical tests, to control and identify the impact of common method biases (Podsakoff et al., 2003). For the design of study's procedures, firstly, the questionnaire hides the introduction of the research purpose and the meaning of the variables to minimize the social desirability bias (Yu et al., 2020a). Secondly, all measurement items were randomly assigned in the questionnaire to control retrieval cues prompted by the question context, and some items were also reversed to examine whether the respondents were responsible for answering. Moreover, this paper promised to protect the anonymity of the respondents and told them that the answers filled in were not right or wrong so that they could reduce their concerns about the survey and answer questions as honestly as possible.

For the post-statistical test, first, following the method used by Liang et al. (2007), we added a common method factor, which incorporated all the principal constructs' indicators. Then, we calculated average indicator's variances, which were substantively explained by the constructs and by the common method factor (CMV). Table 6 indicates that the average substantive variance of indicators (R1)^2^ was 0.640, while the average method-based variance (R2)^2^ was just only 0.007, and the ratio of these two is about 91:1. Likewise, most method factor loadings were also insignificant, suggesting that the method variance was small (Sheng et al., 2020). Second, Harman's single-factor test was used (Podsakoff et al., 2003). The results show that the first factor without rotation can only explain the variance of 19.769% (the total explanatory variance is 68.925%), which is lower than the 50% criterion, showing that the impact of common method variance was not enough to affect results.

**Table 6:** Common method bias analysis (*N* = 254).

**Construct**	**Indicator**	**Substantive**	**(R1)^**2**^**	**Method**	**(R2)^**2**^**
		**factor**		**factor**	
		**loading (R1)**		**loading (R2)**	
Evasive hiding (EH)	EH1	0.785^***^	0.616	0.109	0.012
	EH 2	0.787^***^	0.619	0.009	0.000
	EH 3	0.896^***^	0.803	−0.031	0.001
	EH 4	0.871^***^	0.759	−0.093	0.009
Playing dumb (PD)	PD1	0.827^***^	0.684	0.065	0.004
	PD2	0.843^***^	0.711	0.045	0.002
	PD3	0.846^***^	0.716	−0.019	0.000
	PD4	0.934^***^	0.872	−0.091	0.008
Techno-overload (TE-OVER)	TE-OVER2	0.685^***^	0.469	0.039	0.002
	TE-OVER3	0.820^***^	0.672	−0.013	0.000
	TE-OVER4	0.735^***^	0.540	0.037	0.001
Techno-invasion (TE-INVA)	TE-INVA1	0.822^***^	0.676	0.019	0.000
	TE-INVA2	0.850^***^	0.723	−0.085	0.007
	TE-INVA3	0.857^***^	0.734	−0.053	0.003
	TE-INVA4	0.756^***^	0.572	0.116	0.013
Techno-complexity (TE-COM)	TE-COM2	0.877^***^	0.769	−0.022	0.000
	TE-COM3	0.850^***^	0.723	−0.042	0.002
	TE-COM4	0.764^***^	0.584	−0.060	0.004
	TE-COM5	0.744^***^	0.554	0.120	0.014
Techno-insecurity (TE-INS)	TE-INS1	0.768^***^	0.590	0.015	0.000
	TE-INS2	0.752^***^	0.566	−0.108	0.012
	TE-INS3	0.773^***^	0.598	0.021	0.000
	TE-INS4	0.816^***^	0.666	0.017	0.000
	TE-INS5	0.800^***^	0.640	0.038	0.001
Techno-uncertainty (TE-UNC)	TE-UNC1	0.818^***^	0.669	−0.018	0.000
	TE-UNC2	0.866^***^	0.750	0.045	0.002
	TE-UNC4	0.878^***^	0.771	−0.028	0.001
Work exhaustion (WE)	WE1	0.786^***^	0.618	0.033	0.001
	WE2	0.443^***^	0.196	0.215^*^	0.046
	WE3	0.540^***^	0.292	0.121^*^	0.015
	WE4	0.972^***^	0.945	−0.179	0.032
	WE5	0.905^***^	0.819	−0.099	0.010
Friendship Opportunity (FO)	FO1	0.680^***^	0.462	0.069	0.005
	FO2	0.531^***^	0.282	−0.208^*^	0.043
	FO3	0.754^***^	0.569	0.073	0.005
	FO4	0.805^***^	0.648	0.005	0.000
	FO5	0.726^***^	0.527	0.035	0.001
Friendship Prevalence (FP)	FP1	0.823^***^	0.677	0.007	0.000
	FP2	0.872^***^	0.760	0.036	0.001
	FP3	0.889^***^	0.790	0.041	0.002
	FP4	0.775^***^	0.601	−0.091	0.008
Average			0.640		0.007

* *p < 0.05*;

*** *p < 0.001*.

Hence, given the above procedures' control design and post-statistical tests, we contend that CMV is not a concern in this study, laying a solid foundation for further empirical tests.

### Structural Model Analysis

Before the hypothesis testing, we had used the variance inflation factor (VIF) index to assess the collinearity problem. The results show that the VIF values for the variables, including techno-overload (VIF = 1.510), techno-invasion (VIF = 1.578), techno-complexity (VIF = 1.741), techno-security (VIF = 1.761), techno-uncertainty (VIF = 1.515), and workplace friendship (VIF = 1.279), which all meet the rule of thumb that being smaller than the value of 5 (Hair et al., 2017). Hence, the collinearity is not critical in this study. In the structural model analysis, in order to test whether the path coefficient was significant, this paper used the bootstrap method to repeatedly create 5,000 samples (each sample size is 254) so as to produce the more robust values of standard error (SE). For the result of structural model analysis, if the bias-corrected 95% confidence interval (CI) does not contain zero, it is significant.

#### Direct Effect Analysis

Table 7 shows the structural model outcomes after controlling the influence of demographic variables. The relationships between techno-complexity and work exhaustion (β = 0.140, SE = 0.061, *p* < 0.05), techno-invasion and work exhaustion (β = 0.300, SE = 0.067, *p* < 0.001), techno-insecurity and work exhaustion (β = 0.175, SE = 0.066, *p* < 0.001) are positive and significant. H1b, H1c, and H1d are supported by empirical data. The standardized regression coefficients of techno-overload on work exhaustion (β = −0.035, SE = 0.038, *p* > 0.05), techno-uncertainty on work exhaustion (β = −0.055, SE = 0.042, *p* > 0.05) are not significant; H1a and H1e are not supported. In addition, compared with the relationships of techno-complexity on work exhaustion, and techno-insecurity on work exhaustion, the standardized regression coefficient for the relationship between techno-overload and work exhaustion is the largest (0.300 > 0.175 > 0.140).

**Table 7:** Hypothesized direct effects (*N* = 254).

**Direct effects**	**Coefficient**	**Bootstrap 5,000**		
		**times output**		
		**SE**	***t*-value**	***p*-value**
Techno-complexity−>Work exhaustion	0.140	0.061	2.279	0.023
Techno-invasion−>Work exhaustion	0.300	0.067	4.477	0.000
Techno-insecurity−>Work exhaustion	0.175	0.066	2.637	0.009
Techno-uncertainty−>Work exhaustion	−0.055	0.042	1.312	0.190
Techno-overload−>Work exhaustion	−0.035	0.038	0.935	0.350
Work exhaustion−>Knowledge hiding	0.465	0.067	6.982	0.000
Knowledge hiding−>Evasive hiding (second order construct)	0.916	0.014	64.017	0.000
Knowledge hiding−>Playing dumb (second order construct)	0.921	0.014	66.111	0.000
Techno-complexity−>Knowledge hiding (control)	−0.016	0.045	0.355	0.722
Techno-insecurity−>Knowledge hiding (control)	0.169	0.073	2.319	0.021
Techno-invasion−>Knowledge hiding (control)	−0.024	0.051	0.459	0.646
Techno-overload−>Knowledge hiding (control)	0.015	0.043	0.354	0.724
Techno-uncertainty−>Knowledge hiding (control)	−0.055	0.055	0.991	0.322
Gender−>Knowledge hiding (control)	−0.041	0.041	0.986	0.324
Gender−>Work exhaustion (control)	0.063	0.041	1.528	0.127
Position state2−>Knowledge hiding (control)	−0.107	0.072	1.489	0.137
Position state2->Work exhaustion (control)	0.119	0.062	1.931	0.054
Working years1->Knowledge hiding (control)	0.067	0.058	1.157	0.248
Working years1−>Work exhaustion (control)	0.086	0.054	1.588	0.113
Working years2−>Knowledge hiding (control)	0.078	0.053	1.466	0.143
Working years2−>Work exhaustion (control)	0.064	0.047	1.373	0.170
Education−>Knowledge hiding (control)	0.057	0.039	1.458	0.145
Education->Work exhaustion (control)	−0.049	0.044	1.128	0.260
Age1−>Knowledge hiding (control)	−0.105	0.083	1.262	0.207
Age1−>Work exhaustion (control)	0.067	0.073	0.920	0.358
Age2−>Knowledge hiding (control)	−0.032	0.056	0.567	0.571
Age2−>Work exhaustion (control)	0.134	0.076	1.776	0.076
Position state1−>Knowledge hiding (control)	−0.185	0.080	2.300	0.022
Position state1−>Work exhaustion (control)	0.152	0.070	2.179	0.030

The relationship between work exhaustion and knowledge hiding is positive and significant (β = 0.465, SE = 0.067, *p* < 0.001); H2 is supported. Furthermore, the significantly positive relationships between knowledge hiding and evasive hiding (β = 0.916, SE = 0.014, *p* < 0.001), knowledge hiding and playing dumb (β = 0.921, SE = 0.014, *p* < 0.001) indicate that the second-order construct of knowledge hiding is supported.

#### Indirect Effect Analysis

The mediation effect was examined by Sobel test and bootstrap. Firstly, the Sobel test method shows that the indirect paths of “techno-complexity− >work exhaustion− > knowledge hiding” (Sobel *Z*-value is 2.249, *p* < 0.05), “techno-invasion− >work exhaustion− >knowledge hiding” (Sobel *Z*-value is 3.739, *p* < 0.001), “techno-insecurity− >work exhaustion− > knowledge hiding” (Sobel *Z*-value is 2.386, *p* < 0.05) are significant. H3b, H3c, and H3d are supported. However, “techno-overload− >work exhaustion− > knowledge hiding” is not significant (Sobel *Z*-value is 0.938, *p* > 0.05), and “techno-uncertainty− >work exhaustion− >knowledge hiding” (Sobel *Z*-value is 1.287, *p* > 0.05) is also not significant. Since the premise assumption of the Sobel test method is based on the normal distribution, it usually does not conform to the normal distribution after multiplying the path coefficients (Hair et al., 2017). Therefore, this study used the bootstrap method to further test the robustness of mediation effect.

Secondly, 5,000 bootstrap samples are drawn from the original sample (254 observations are drawn each time) with replacement in bootstrapping. Replacement indicates that, each time, an observation is drawn at random from the sampling population and is returned to the sampling population before the next observation is drawn (Hair et al., 2017). The Smart-PLS software uses the 5,000 bootstrap samples to estimate the indirect effects. The estimates of the 5,000 coefficients form a bootstrap distribution, which can be viewed as an approximation of the sampling distribution (Hair et al., 2017). Based on this distribution, we can obtain standard error and test the significance of the indirect effect.

Table 8 shows that the indirect paths of “techno-invasion− >work exhaustion− >knowledge hiding” (indirect effect, 0.139, bias-corrected 95% CI = 0.069 to 0.220, not including zero), “techno-complexity− >work exhaustion− > knowledge hiding” (indirect effect, 0.065, bias-corrected 95% CI = 0.016 to 0.146, not including zero), “techno-insecurity− >work exhaustion− >knowledge hiding” (indirect effect, 0.081, bias-corrected 95% CI = 0.020 to 0.156, not including zero) are significant. H3b, H3c, and H3d are supported again. However, “techno-uncertainty− >work exhaustion− >knowledge hiding” is not significant (indirect effect, −0.026, bias-corrected 95% CI = −0.093 to 0.017, including zero), and “techno-overload− >work exhaustion− >knowledge hiding” (indirect effect, −0.016, bias-corrected 95% CI = −0.087 to 0.033, including zero) is not significant. H3a and H3e are not supported.

**Table 8:** The indirect effect of hypothesized paths (*N* = 254).

**Paths**	**Estimate**	**Bootstrap 5,000 times**		
		**bias-corrected intervals**		
		**95% lower**	**95% upper**	**Significance**
Techno-invasion− >Work exhaustion− >Knowledge hiding	0.139	0.069	0.220	Yes
Techno-complexity− >Work exhaustion− >Knowledge hiding	0.065	0.016	0.142	Yes
Techno-insecurity− >Work exhaustion− >Knowledge hiding	0.081	0.020	0.156	Yes
Techno-uncertainty− >Work exhaustion− >Knowledge hiding	−0.026	−0.093	0.017	No
Techno-overload− >Work exhaustion− >Knowledge hiding	−0.016	−0.087	0.033	No

#### Moderation of Workplace Friendship

**Figure 3:**
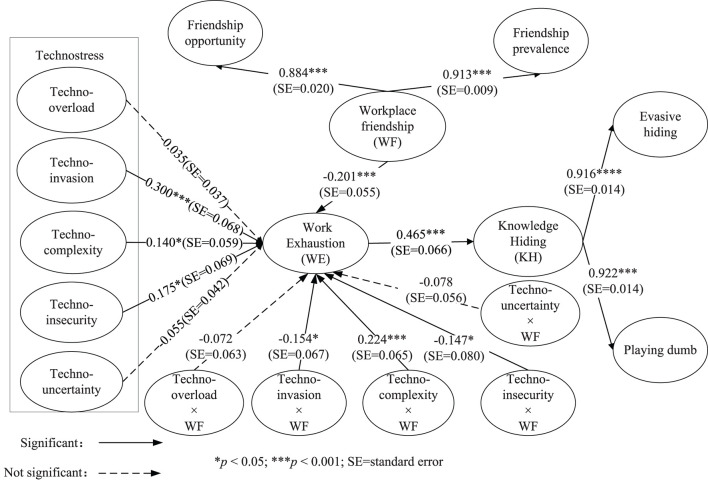
The moderation analysis for workplace friendship (*N* = 254).

The orthogonalizing approach was used for creating interaction terms when conducting the moderation analysis of workplace friendship. Compared with the product indicator approach and the two-stage approach, the orthogonalizing approach can minimize the estimation bias in terms of point accuracy, and it can yield high prediction accuracy (Hair et al., 2017). Figure 3 shows that the interaction term of techno-invasion and workplace friendship on work exhaustion is negative and significant (β = −0.154, SE = 0.067, *p* < 0.05). H4b is supported. The interaction term of techno-insecurity and workplace friendship on work exhaustion is also negative and significant (β = −0.147, SE = 0.080, *p* < 0.05). H4d is supported. However, the interaction term of techno-complexity and workplace friendship on work exhaustion is positive and significant (β = 0.224, SE = 0.065, *p* < 0.001), which is opposite to the original hypothesis (from a negative relationship to positive). H4c is not supported. The interaction term of techno-overload and workplace friendship on work exhaustion is negative but not significant (β = −0.072, SE = 0.063, *p* > 0.05). H4a is not supported. The interaction term of techno-uncertainty and workplace friendship on work exhaustion is negative but not significant (β = −0.078, SE = 0.056, *p* > 0.05). H4e is not supported.

Furthermore, in order to evaluate the moderation effect, this study used the *f*^2^ index in Smart-PLS for the effect size of workplace friendship moderation (Hair et al., 2017). The specific calculation formula is as follows:


$$f^{2}=\frac{R^{2}\text{included}-R^{2}\text{excluded}}{1-R^{2}\text{included}}$$

Note(s): Where *R*^2^ included and *R*^2^ excluded are the *R*^2^ values of the endogenous latent variable when the interaction term of the moderator model is included in or excluded from the PLS path model.

The effect size of workplace friendship for the relationships that technostress (including five dimensions) on work exhaustion are evaluated. When the interaction term of techno-invasion and workplace friendship is included in the model, the *R*^2^ for work exhaustion is 0.463, while, after excluding the interaction term, the *R*^2^ for work exhaustion is reduced to 0.448. According to the above formula calculation, the effect size value for the interaction term of techno-invasion and workplace friendship on work exhaustion is 0.028.

Following the same steps, when the interaction term of techno-insecurity and workplace friendship is included in or excluded from the PLS path model, the *R*^2^ of work exhaustion is 0.463 and 0.449, respectively. And the value of effect size for the interaction term of techno-insecurity and workplace friendship on work exhaustion is 0.026. The *R*^2^ of work exhaustion is 0.463 and 0.425 when including or excluding the interaction term of techno-complexity and workplace friendship, and its effect size is 0.071. When the interaction term of techno-overload and workplace friendship is included in and excluded from the model, the *R*^2^ of work exhaustion is 0.463 and 0.460, respectively, and its effect size is 0.006. The *R*^2^ of work exhaustion is 0.463 and 0.459, respectively, when including or excluding the interaction term of techno-uncertainty and workplace friendship, and its effect size is 0.007.

According to the criterion of Kenny and Judd (2019), the values of 0.005, 0.01, and 0.025, respectively, represent the small, medium, and large effect size. Then the moderation of workplace friendship on the relationships between the two aspects of technostress (techno-invasion and techno-insecurity) and work exhaustion are at a large level, which further supports H4b and H4d. Although the effect size of workplace friendship for the relationship between techno-complexity and work exhaustion is at a large level, it is in the opposite direction to the hypothesis. In addition, the moderation effect size of workplace friendship for the relationships between the two aspects of technostress (techno-overload and techno-uncertainty) and work exhaustion is at the low level. H4a and H4e are not supported again.

**Figure 4:**
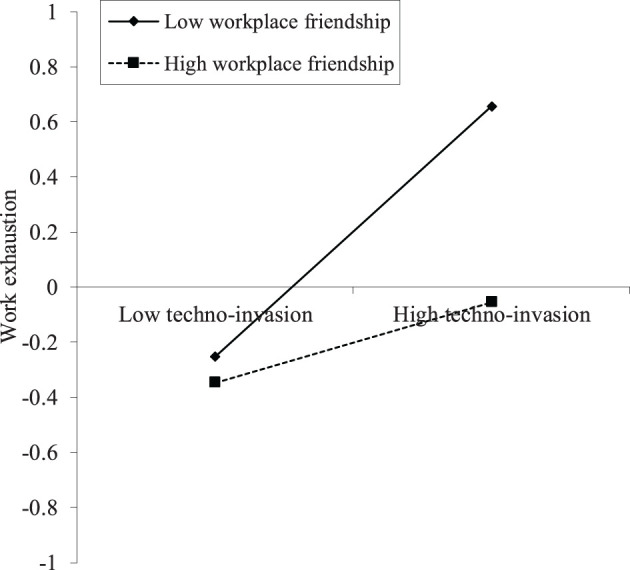
The interaction of techno-invasion and WF on work exhaustion (*N* = 254).

**Figure 5:**
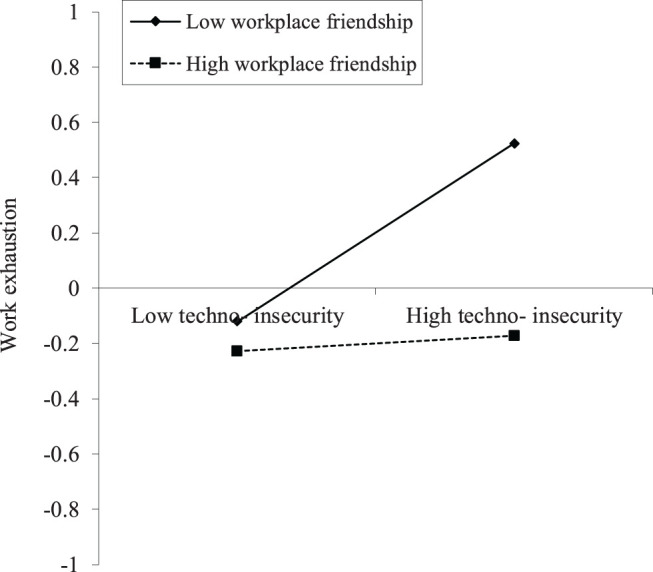
The interaction of techno-insecurity and WF on work exhaustion (*N* = 254).

The slope plots are used to illustrate the results of the supported moderation hypothesis. Figure 4 indicates that workplace friendship has a negative moderation on the relationship between techno-invasion and work exhaustion, where the x-axis represents techno-invasion and the y-axis work exhaustion. The relationship between techno-invasion and work exhaustion becomes stronger with low levels of workplace friendship. For high levels of workplace friendship, the slope is much flatter. Hence, with high levels of the workplace friendship, the relationship between techno-invasion and work exhaustion becomes weaker. Following the same logic, Figure 5 shows that, with high levels of the workplace friendship, the relationship between techno-insecurity and work exhaustion becomes weaker. The results of Figures 4, 5 support the H4b and H4d again.

Furthermore, this study examined the moderation effect of workplace friendship on the mediation effects of work exhaustion between the two aspects of technostress (techno-invasion and techno-insecurity) and knowledge hiding. The study adopted the index of moderated mediation suggested by Hayes (2015). The moderated mediation effect (denoted as ω) can be written as follows:


(1) $$\begin{array}{r} \text{ω}=\left(a_{1\text{or}2}+a_{3}WF\right)b\; \end{array}$$

(2) $$\begin{array}{r} \text{ω}=a_{1\text{or}2}b+a_{3}WFb \end{array}$$

Note(s): WF = workplace friendship.

In the above equations, a_1or2_ (techno-invasion/techno-insecurity on work exhaustion), b (work exhaustion on knowledge hiding), and a_3_ (the interaction term on work exhaustion) are estimated coefficients. Hayes (2015) calls a_3_b as the index of moderated mediation, which “is a quantification of the effect of (the moderator) on the indirect effect of (the predictor) on (the outcome variable) through (the mediator).” In order to test the index significance, the bootstrapping was used to generate a bootstrap confidence interval. Table 9 shows that the index value (a_3_b = 0.144) for the indirect effect of “techno-invasion− >WE− >KH” is significant at 0.05. And the index value (a_3_b = 0.136) for the indirect effect of “techno-insecurity− >WE− >KH” is significant at 0.1. Specifically, Table 9 shows that, when the workplace friendship is high (+ 1SD), the indirect effect that techno-invasion on knowledge hiding through work exhaustion is not significant (β = 0.068, SE = 0.047, *p* > 0.1), and the indirect effect of techno-insecurity on knowledge hiding through work exhaustion is likewise not significant (β = 0.013, SE = 0.055, *p* > 0.1), which indicates that workplace friendship can weaken the mediating effect of work exhaustion between the two aspects of technostress (techno-invasion and techno-insecurity) and knowledge hiding. When workplace friendship is high, the indirect effects of work exhaustion between the two aspects of technostress (techno-invasion and techno-insecurity) and knowledge hiding are not significant.

**Table 9:** Moderated mediation test (*N* = 254).

**Indirect effect**	**Index for moderated mediation**	**SE**	***p***	**Significance**
Techno-invasion− >WE− >KH	0.144	0.066	<0.05	Yes
Techno-insecurity− >WE− >KH	0.136	0.075	<0.1	Yes
Moderator: WF	Indirect effect	SE	*p*	Significance
+1SD	Techno-invasion− >WE− >KH = 0.068	0.047	>0.1	No
−1SD	Techno-invasion− >WE− >KH = 0.212	0.052	<0.001	Yes
+1SD	Techno-insecurity− >WE− >KH = 0.013	0.055	>0.1	No
−1SD	Techno-insecurity− >WE− >KH = 0.149	0.043	<0.001	Yes

*WE, work exhaustion; KH, knowledge hiding; WF, workplace friendship; SE, standard error; SD, standard deviation*.

## Conclusion and Implications

### Findings and Discussions

Technostress as an antecedent factor exploring knowledge hiding continues to be seldomly discussed in the digital era. Based on the job demand-resource theory, this article introduces work exhaustion as a mediator variable and constructs a model that the five sub-dimensions of technostress (i.e., overload, invasion, complexity, insecurity, and uncertainty) affect knowledge hiding for the R&D employees. Similarly, this study analyzes the moderation of workplace friendship as the resource buffering effect. On the basis of the 254 questionnaires of the two-stage survey, it empirically tests the research model. The findings are as follows.

Techno-invasion, techno-insecurity, and techno-complexity have significant positive effects on work exhaustion, and techno-invasion has the greatest effect. However, techno-overload and techno-uncertainty have no significant relationship with work exhaustion based on our empirical results. These findings suggest that not all five dimensions of technostress will lead to work exhaustion but when the situations where the R&D employees' personal and professional lives are blurred due to digital technology, where the R&D employees feel they may lose their own work replaced by technology, and where the R&D employees are unfamiliar with digital technology and need to spend a lot of time and energy for learning. And when digital technology prompts R&D employees to work anytime, anywhere without a dividing line between work and family life is the most likely to cause employees to suffer from work exhaustion (Bauwens et al., 2021). The possible reason why the relationships between the two aspects of technostress (techno-overload, techno-uncertainty) and work exhaustion are insignificant is as follows. Techno-overload emphasizes that employees need to deal with more work information and conduct multiple works simultaneously (Ragu-Nathan et al., 2008). Considering that the R&D employees are engaged in knowledge-intensive work, obtaining further information from different sources can easily promote their confidence and self-efficacy for their R&D work (Liao et al., 2021). In the current era of “information explosion” (Alzahrani and Seth, 2021), employees may be more adaptable to the stress of techno-overload and will not significantly experience work exhaustion. Moreover, the constant change/upgrade in digital technologies creates uncertainty, and it has become normal in today's rapidly changing times (Teece et al., 2016). Specifically, digital technology changes more and more rapidly, especially for the electronic information equipment, which launches a new version every year. People are adapting to the rapidly changing world of technology, and, when the R&D employees feel technical uncertainty, it results in imperceptible work exhaustion.

Work exhaustion plays a mediating role in the relationships between the three aspects of technostress (techno-invasion, techno-insecurity, techno-complexity) and knowledge hiding. However, its mediating effects are insignificant in the relationships between the two aspects of technostress (techno-overload and techno-uncertainty) and knowledge hiding. These findings suggest that the three dimensions of technostress, including invasion, insecurity, and complexity, will affect the R&D employees' knowledge hiding through the internal mechanism of work exhaustion, supporting the core opinion of the JD-R theory that “higher job demands are likely to exhaust employees' resources and lead to a state of exhaustion, therefore resulting in negative consequences” (Bakker and Demerouti, 2017). However, the result that techno-overload and techno-uncertainty cannot affect knowledge hiding through work exhaustion again supports the result of hypothesis 1, indicating that not all five dimensions of technostress will affect R&D knowledge hiding through work exhaustion but only three dimensions of techno-invasion, techno-insecurity, and techno-complexity.

Workplace friendship negatively moderates the relationships between the two aspects of technostress (techno-invasion and techno-insecurity) and work exhaustion, leading to less knowledge hiding. Nonetheless, its negative moderation for the relationships between the two aspects of technostress (techno-overload and techno-uncertainty) and work exhaustion are insignificant. Moreover, the empirical results show that workplace friendship positively moderates the relationship between techno-complexity and work exhaustion. Some findings support the “resource buffering effect” derived from the JD-R theory, which indicates that we should pay extra attention to high workplace friendship when weakening the relationships between the two aspects of technostress (techno-invasion and techno-insecurity) and work exhaustion, in order to reduce knowledge hiding. The insignificant moderation of workplace friendship for the relationships between the two aspects of technostress (techno-overload and techno-uncertainty) and work exhaustion further support the results in hypothesis 1. However, empirical analysis yielded a surprising result that workplace friendship positively moderates the relationship between techno-complexity and work exhaustion. This finding is contrary to the “resource buffering” effect of the JD-R theory. The possible reason is that although the benefits of workplace friendship are many, the challenges also exist, including devoting time to the friendship and distraction from work (Morrison and Nolan, 2007; Hood et al., 2017). In a high workplace friendship environment, the better relationships among colleagues can largely cause R&D employees to waste a considerable amount of time building relationships, leading to less time to learn the complex technology, compelling R&D employees to face more technical complexity which is likely to increase their work exhaustion.

### Theoretical Implications

This paper expounds on the theoretical contribution, following the logic of the theoretical composition, including construct, relationship, mechanism, and context (Colquitt and Zapata-Phelan, 2007).

We introduce the technostress construct into the field of R&D employee knowledge hiding, adding a new insight into the antecedents of knowledge hiding. Through a systematic literature review, we found that, although certain literature explores the antecedents of knowledge hiding from the individual-level factors, including demographic variables, personality traits (Anaza and Nowlin, 2017; Pan et al., 2018), and cognitive and psychological aspects (Wu, 2020; He et al., 2021b), the existing research scarcely explores knowledge hiding from the perspective of digital technology stress. On the basis of the perspective of technical stress, this study creatively introduces the concept of technostress, explores its influence on knowledge hiding, and carefully analyzes the influence of five sub-dimensions (i.e., overload, invasion, complexity, insecurity, and uncertainty). The concept of technostress was introduced into the field of knowledge hiding antecedent variables, theoretically increasing the formation and explanation of the knowledge hiding of the R&D employees in the context of the digital age and better helping people understand the negative role of digital technology in knowledge hiding. Our work is one of the pioneering studies in the analysis of knowledge hiding in the digital age because the most recent studies have continued to explore knowledge hiding in relation to classic organizational phenomena, occurring regularly in the context of the pre-digital era (Arias-Perez and Velez-Jaramillo, 2021), such as distrust (Xiong et al., 2021) or workplace gossip and bullying (Yao et al., 2020a,b).

The individual effects of five technostress dimensions on work exhaustion are analyzed, making some theoretical contributions from the relationship element of theory. Although a few of the existing studies have explored the relationship between technostress and work burnout (Yener et al., 2020), relatively few studies exist on a detailed analysis of techno-invasion, techno-insecurity, techno-complexity, techno-overload, and techno-uncertainty on work exhaustion. The extant literature has described technostress as a second-order construct in an aggregated form without the individual impact of different dimensions (Yener et al., 2020; Harris et al., 2021). This study rigorously analyzes the relationship among five dimensions of technostress and work exhaustion and finds that the relationship of techno-invasion on work exhaustion has the largest effect, which can help people understand the difference in the impact of different dimensions of technostress for work exhaustion.

We studied the internal process that different dimensions of technostress have impacts on knowledge hiding from the perspective of work exhaustion, further elaborating the mechanism that technostress increases knowledge hiding. The existing research explores the internal mechanism of knowledge hiding from the perspective of Islamic work ethics (Khalid et al., 2018), moral disengagement (Zhao and Xia, 2019), and psychological contract breach (Ghani et al., 2020). In our study, the influence mechanism of “technostress (five dimensions)-work exhaustion-knowledge hiding” indicates the formation of knowledge hiding for the R&D employees and can better help people understand the inner process that technostress (five dimensions) on knowledge hiding. We also responded to the research direction of “more work is needed to provide comprehensive studies on the generating mechanisms for knowledge hiding” proposed by He et al. (2021a).

The workplace friendship contextual exploration can extend the understanding on how to buffer the negative effect of technostress. Existing research focuses on the perspective of technological self-efficacy (Tarafdar et al., 2015), psychological entitlement (Harris et al., 2021), and time management (Yener et al., 2020) when exploring the moderation for the relationship between technostress and outcome variables. However, prior studies lack consideration from the perspective of workplace friendship. The empirical results find that workplace friendship can significantly and negatively moderate the relationships between the two aspects of technostress (techno-invasion, techno-insecurity) and work exhaustion, leading to less knowledge-hiding behavior. We apply the JD-R theory to the contextual effect of digital technology stress on knowledge hiding, helping people understand how to reduce the impact of digital technology stress on knowledge hiding and addressing the call that “more work is needed to study the respective coping strategies of knowledge hiding” (He et al., 2021a).

### Practical Implications

The study provides the R&D managers with an understanding of the impact of technostress on knowledge hiding. Most managers are concentrated on understanding what digital technologies can do *for* you, but they pay little attention to considering what digital technologies can do *to* you. In today's world, the outbreak of COVID-19 accelerates the digital transformation process. Organizations are more likely to implement, upgrade, and assimilate digital technologies more than ever, and employees have to deal with the effect of technostress. Technostress plays a critical role in driving knowledge hiding, and the R&D managers should endeavor to relieve the negative effect by implementing relevant training and support.

First, the R&D department managers ought to be attuned to the factors that induce the three kinds of technostress, including techno-invasion, techno-insecurity, and techno-complexity, to ease the stress on the employees because the empirical results show that they will significantly affect the knowledge hiding of the R&D employees through work exhaustion. The R&D department managers need to pay special attention to the invasion of digital technology for the lives of the R&D employees. By developing workplace policies, the R&D department can allow employees not to process work information immediately during off-duty hours *via* digital technology. In addition, the R&D department should also conduct psychological counseling for the employees to let them realize that digital technology will not completely replace their jobs. The R&D employees can better enhance their competitiveness and be able to meet the job requirements in the digital age by earnestly learning digital technology knowledge. Furthermore, before adopting new technologies, the R&D department should develop the R&D employees' technical competence by offering technical training so that the employees can fully understand the changes and impacts of new digital technologies on their work. After grasping the relevant knowledge of digital technology, the R&D employees can psychologically weaken the sense of complexity about new technologies.

Second, the R&D department needs to pay special attention to the work exhaustion among the R&D employees, because it plays a mediator role in the relationships between the three aspects of technostress (techno-invasion, techno-insecurity, techno-complexity) and knowledge hiding. If the R&D employees show symptoms of work exhaustion, the R&D department needs immediate psychological counseling to get rid of it as much as possible to better reduce the behavior of knowledge hiding.

Third, the R&D department needs to create a good atmosphere of workplace friendship. This study found that workplace friendship can significantly reduce the relationships between the two aspects of technostress (techno-invasion and techno-insecurity) and work exhaustion, leading to less knowledge hiding. These findings show that the R&D department should create a good atmosphere for the R&D employees to establish better workplace friendship and weaken the influence of techno-invasion and techno-insecurity on knowledge hiding. However, the establishment of workplace friendship environment cannot reduce the impact of techno-complexity on work exhaustion. This finding suggests that the R&D department should reduce the generation of techno-complexity through technology training as described above to reduce its negative impact.

### Future Research Direction

Future research needs to explore the relationships between the two aspects of technostress (techno-overload and techno-uncertainty) and work exhaustion, and the moderating effect of workplace friendship on the relationships between the three aspects of technostress (techno-overload, techno-uncertainty, and techno-complexity) and work exhaustion. Based on the JD-R theory, this paper argues that techno-overload and techno-uncertainty affect work exhaustion significantly, and workplace friendship negatively moderates the relationships between the three aspects of technostress (techno-overload, techno-uncertainty, and techno-complexity) and work exhaustion. However, the empirical results do not support the theoretical hypothesis results. Although this paper tentatively gives certain explanations, whether it is a problem from the theory or the empirical test process can not be answered by only one empirical test. Thus, another empirical tests will be needed in the future.

## Data Availability Statement

The raw data supporting the conclusions of this article will be made available by the authors, without undue reservation.

## Author Contributions

ZZ and BY conceived and designed the work and analyzed and interpreted the data. CY collected the data. ZQ and BY drafted the article. ZZ, CY, and HZ are responsible for the modifications. All authors contributed to the article and approved the submitted version.

## Funding

This research was supported by the Major Program of National Fund of Philosophy and Social Science of China (No. 18ZDA062).

## Conflict of Interest

The authors declare that the research was conducted in the absence of any commercial or financial relationships that could be construed as a potential conflict of interest.

## Publisher's Note

All claims expressed in this article are solely those of the authors and do not necessarily represent those of their affiliated organizations, or those of the publisher, the editors and the reviewers. Any product that may be evaluated in this article, or claim that may be made by its manufacturer, is not guaranteed or endorsed by the publisher.
